# CT findings of lung injury during breast cancer treatment

**DOI:** 10.1007/s12282-025-01816-1

**Published:** 2025-12-19

**Authors:** Ken Yamaguchi, Ryoko Egashira, Takahiko Nakazono, Yutaka Yoshinaga, Koichi Baba, Masako Kataoka, Hidetake Yabuuchi, Takatoshi Aoki, Osamu Togao

**Affiliations:** 1 Department of Radiology, Faculty of Medicine, Saga University, 5-1-1 Nabeshima, Saga, 849-8501 Japan; 2 Satsuma Lab, Hawkes Institute, University College London, 90 High Holborn, London, WC1V 6BH UK; 3 Department of Radiology, Saga-ken Medical Center Koseikan, 400 Nakabaru, Kasemachi, Saga City, 840-8571 Japan; 4 Department of Surgery, Faculty of Medicine, Saga University, 5-1-1 Nabeshima, Saga, 849-8501 Japan; 5 Preemptive Medicine and Lifestyle-related Disease Research Center, Kyoto University Hospital, 53 Kawahara-cho, Shogoin, Sakyo-ku, Kyoto, 606- 8507 Japan; 6 Department of Health Sciences, Kyushu University Graduate School of Medical Sciences, 3-1-1 Maidashi, Higashi-ku, Fukuoka, 812-8582 Japan; 7 Department of Radiology, University of Occupational and Environmental Health, 1-1 Iseigaoka, Yahatanichi-ku, Kitakyushu, 807-8555 Japan

**Keywords:** Breast cancer, Drug-induced lung injury, Radiation-induced lung injury, CT

## Abstract

While breast cancer treatment outcomes have improved significantly through multidisciplinary approaches including surgery, chemotherapy, and radiation therapy, the incidence of non-neoplastic pulmonary complications has also increased. Accurate interpretation of chest imaging is essential for managing these adverse events. This review outlines the major radiological findings of pulmonary injury during breast cancer treatment, focusing on two primary categories: drug-associated interstitial lung disease (DILD) and radiotherapy-associated lung injury (RLI). Regarding DILD, its clinical features, risk factors, differential diagnosis, and diverse patterns on high-resolution CT (HRCT) are described. The lung injury characteristics associated with specific drugs used in breast cancer treatment are also examined, placing particular emphasis on clinically important agents like trastuzumab deruxtecan. Regarding RLI, the pathophysiology (including acute radiation pneumonitis and chronic radiation fibrosis), relevant risk factors, and typical CT findings localized to the radiation field are discussed. Atypical manifestations are also addressed, such as radiation-induced organizing pneumonia (OP) outside the radiation field and the unique phenomenon of radiation recall pneumonitis. These complications can present with symptoms and imaging findings that mimic one another as well as other conditions, such as infections and lymphangitic carcinomatosis. This review aims to facilitate the timely and accurate differential diagnosis of pulmonary complications in breast cancer patients, thereby guiding appropriate therapeutic strategies and enhancing patient safety.

## Introduction

Breast cancer is the most common cancer in women worldwide, and its treatment has improved significantly with advances in multidisciplinary treatment [1]. In addition to surgical treatment, drug therapy (chemotherapy, hormonal therapy, molecular targeted therapy, immunotherapy) and radiation therapy are combined according to the pathology of each individual patient. While these intensive treatments contribute greatly to improving survival outcomes, they can also cause various adverse events. Among them, pulmonary complications require special attention because they can directly affect the patient’s quality of life (QOL) and prognosis.

Thoracic image abnormalities encountered during breast cancer treatment include recurrence and metastasis of the original disease (pulmonary metastasis, pleural metastasis, carcinomatous lymphangitis, bone metastasis, etc.), as well as many non-neoplastic pathologies that are caused by treatment or increase in risk during treatment. Representative examples of the latter are drug-associated interstitial lung injury (DILD) [2] and radiotherapy-associated lung injury (RLI) [3]. These conditions are sometimes difficult to distinguish from infections, and the imaging findings can be similar to each other, so their diagnosis requires careful evaluation of the treatment history and imaging findings.

This article provides a comprehensive review of the chest imaging findings related to treatment that clinicians encounter during the treatment of breast cancer.

## Drug-associated interstitial lung disease (DILD)

Breast cancer treatment strategies have undergone dramatic advancements over the past few decades. In addition to conventional cytotoxic agents and endocrine therapies, the advent of molecularly targeted therapies that target specific molecular pathways and immune checkpoint inhibitors (ICIs) that release the brakes on the immune system has dramatically improved treatment outcomes [4]. However, these therapeutic advances have introduced new adverse event profiles, among which drug-associated interstitial lung disease (DILD) has become recognized as a significant clinical challenge [5]. The clinical presentation of DILD is extremely diverse, ranging from mild cases with only imaging abnormalities and no symptoms to severe cases that progress rapidly to fatal respiratory failure [5]. Severe cases may necessitate the interruption or permanent discontinuation of the causative drug, which can have a major impact on the original cancer treatment plan. Therefore, early detection and appropriate management of DILD are essential for improving the prognosis of breast cancer patients.

### Clinical symptoms, risk factors, and serum biomarkers of DILD

The diagnosis of DILD begins with suspicion of the condition. Its clinical symptoms are nonspecific, and a multifaceted evaluation is necessary to reach a definitive diagnosis.

#### Clinical symptoms

The clinical symptoms of DILD are extremely nonspecific, often presenting with symptoms such as dry cough, dyspnea on exertion, fever, and hypoxemia. These symptoms are similar to those of infections or cancer, making diagnosis difficult. The timing of onset al.so varies by drug, ranging from acute forms that develop within days of starting administration to chronic forms that develop after several months or more [6].

#### Risk factors

Patient-related factors include advanced age, pre-existing interstitial lung disease (ILD), smoking history (especially with a high pack-year index), and Asian ethnicity for certain drugs (e.g., Trastuzumab deruxtecan) [7]. Treatment-related factors include high-cumulative doses of certain drugs (e.g., bleomycin), combination with other drugs with pulmonary toxicity, and a history of chest radiation therapy (concurrent or sequential) [8].

#### Serum biomarkers

Although no specific biomarkers have been established, several serum markers including Lactate Dehydrogenase (LDH), Krebs von den Lungen-6 (KL-6) and Surfactant Protein-D (SP-D) are used as aids in diagnosis and severity assessment [5, 9].

Generally, the severity of the lesion is classified from Grade 1 to 5 by the Common Terminology Criteria for Adverse Events (CTCAE) grade [6, 10]. In CTCAE grade, Grade 1 is asymptomatic, and Grade ≥ 2 events are symptomatic. Grade 4 is life threatening needs urgent intervention and grade 5 is death related to adverse events.

### The role of imaging and major CT patterns of DILD

Not only lung, CT images are useful for evaluating drug-associated injury in various organs, including the liver and pancreas [11–13]. In lung, imaging, especially high-resolution CT (HRCT), plays a central role in the detection, pattern classification, severity assessment, and treatment response evaluation of DILD. To detect early stage of the lesion, HRCT is necessary. Volumetric CT images of both lungs with a slice thickness of 1.25 mm or less are recommended, and these can be reconstructed from a standard chest CT acquisition. DILD often shows bilateral ground-glass opacity (GGO) and consolidation. When widespread or bilateral multiple GGOs or consolidations appears in patients undergoing drug therapy, the possibility of DILD should always be considered.

GGO is an area of increased attenuation that does not completely obscure the underlying vascular structures, and consolidation is denser attenuation with complete obscuration of the underlying vessels [14]. There are no specific imaging findings for DILD, and the same drug can present with various patterns. However, recognizing various imaging patterns is extremely important for estimating the histopathological findings and, consequently, predicting the prognosis [6].

**Fig. 1:**
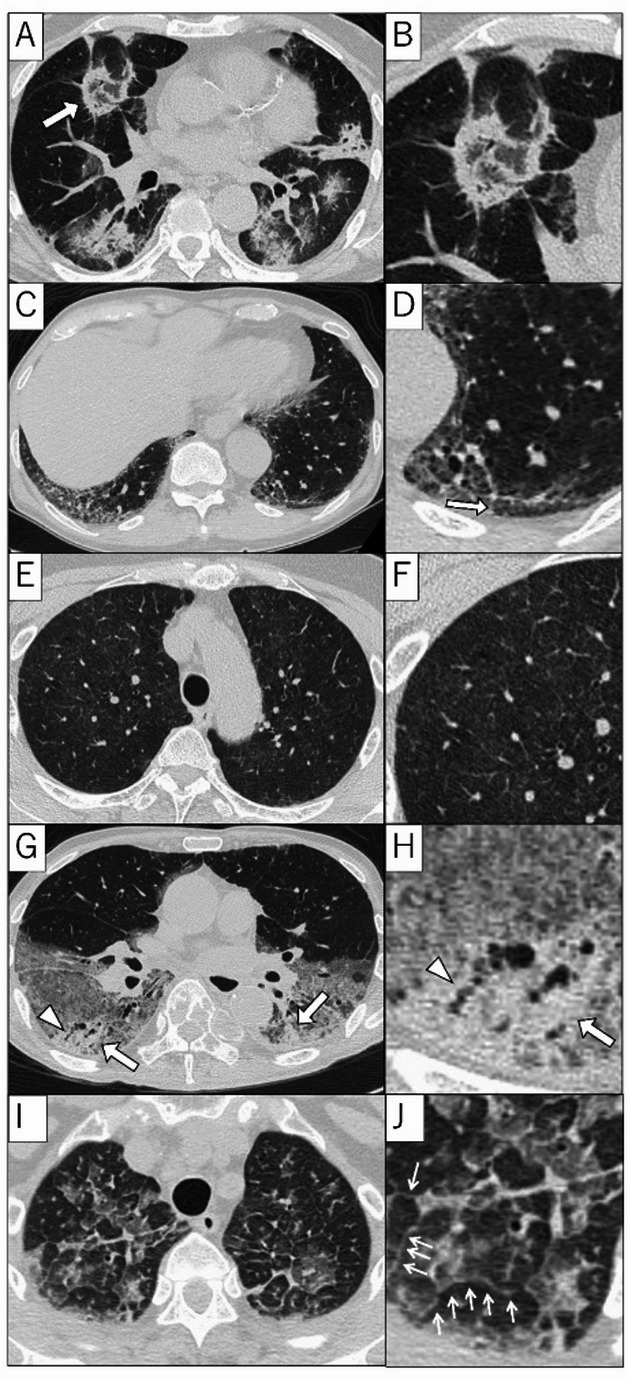
Representative high-resolution CT (HRCT) patterns of Drug-induced lung injury. **A**, **B** Organizing Pneumonia (OP) pattern. Multiple ground-glass opacities (GGOs) and consolidation are seen. Areas with GGO surrounded by a rim-like consolidation (reversed-halo sign, arrow, **A**) are also seen. **C** Nonspecific Interstitial Pneumonia (NSIP) Pattern. There are GGOs and mild retraction with peripheral predominance on the HRCT. The subpleural regions are relatively spared (subpleural sparing). **D** Enlarged image of subpleural sparing (arrow). **E** Hypersensitivity Pneumonitis (HP) Pattern. Bilateral diffuse, ill-defined centrilobular nodules/ GGO are seen on HRCT. **F** Enlarged image of ill-defined nodules/ GGO. **G** Diffuse Alveolar Damage (DAD) Pattern. HRCT image shows extensive GGOs and consolidations (arrows) with dependent zone predominance. Traction bronchiectasis (arrowhead) is also seen. **H** Enlarged image of consolidation (arrow) and traction bronchiectasis (arrowhead). **I** Acute Eosinophilic Pneumonia (AEP) pattern. Bilateral diffuse GGO and interlobular septal thickenings are seen on HECT. **J** Enlarged image of GGO and interlobular septal thickenings (arrow)

There are several major clinically important CT patterns (Fig. 1).

#### Organizing pneumonia (OP) pattern (Fig. 1A and B)

Characterized by patchy, peripherally dominant, or peribronchovascular distributed consolidation and GGO. Lesions are often seen bilaterally. The “reversed halo sign” (atoll sign), where the center of the opacity is GGO and the periphery is rimmed by consolidation, is a characteristic finding but not highly specific. It is generally responsive to steroids and has a relatively good prognosis [6, 15, 16].

#### Nonspecific interstitial pneumonia (NSIP) pattern (Fig. 1C and D)

Characterized by bilaterally symmetric, lower-lobe dominant GGO and reticular opacities. Subpleural sparing can also be seen. DILD often follows a subacute or acute course, and therefore, fibrotic findings such as traction bronchiectasis/bronchioloectasis, or architectural distortion are usually not prominent. However, DILD may occasionally develop insidiously, or in some cases, an initially consolidation-dominant OP-like pattern may progress over time, eventually leading to marked fibrosis [6, 15–17].

#### Hypersensitivity pneumonitis (HP) pattern (Fig. 1E and F)

Characterized by ill-defined centrilobular nodules or GGO. Sometimes, mosaic attenuation can be seen [6, 15–17].

#### Diffuse alveolar damage (DAD) pattern (Fig. 1G and H)

DAD pattern often corresponds to the acute respiratory distress syndrome (ARDS). HRCT shows extensive bilateral GGO and dependent consolidation. Traction bronchiectasis in the earlier stage could be a risk factor of poorer prognosis. DAD pattern is consistently associated with a rapid clinical course and high mortality, regardless of the causative drug, and has an extremely poor prognosis [6, 15–17].

#### Acute eosinophilic pneumonia (AEP) pattern (Fig. 1I and J)

Characterized by diffuse GGO and consolidation. Interlobular septal thickenings are also seen [18].

A summary of major CT patterns of DILD is shown in Table 1.

**Table 1:** Summary of major CT patterns of DILD

CT patten	Findings	Associated drugs	Differential diagnosiss
OP pattern	Bilatelally, patchy, peripherally dominant, or peribronchovascular distributed consolidation and GGO Reversed halo	Abemaciclib, Ribociclib, Evelorimus, T-DXd, Docetaxel, Epirubicin, Pembrolizumab, Atezolizumab	Infectious pneumonia, Chronic EP
NSIP pattern	Bilaterally symmetric, lower-lobe dominant GGO and reticular opacities Subpleural sparing	Evelorimus, T-DXd, Atezolizumab	Idiopathic NSIP
HP pattern	Ill-defined centrilobular nodules or GGO	Evelorimus, T-DXd, Epirubicin	Pneumocystis pneumonia, Alveolar hemorrhage
DAD pattern	Extensive bilateral GGO and dependent consolidation Traction bronchiectasis	Abemaciclib, T-DXd, Pembrolizumab	Acute exacerbation of existing interstitial pneumonia
AEP pattern	Bilateral diffuse GGO and consolidation Interlobular septal thickening	Tamoxifen	Non-cardiogenic pulmonary edema, Lymphangitic carcinomatosis, Lymphoproliferative disorder

### Differential diagnosis

The diagnosis of DILD is made by excluding other diseases with similar clinical and imaging findings, which must be carefully differentiated [6]. The following diseases are particularly important to consider in cancer patients.

#### Infectious pneumonia

**Fig. 2:**
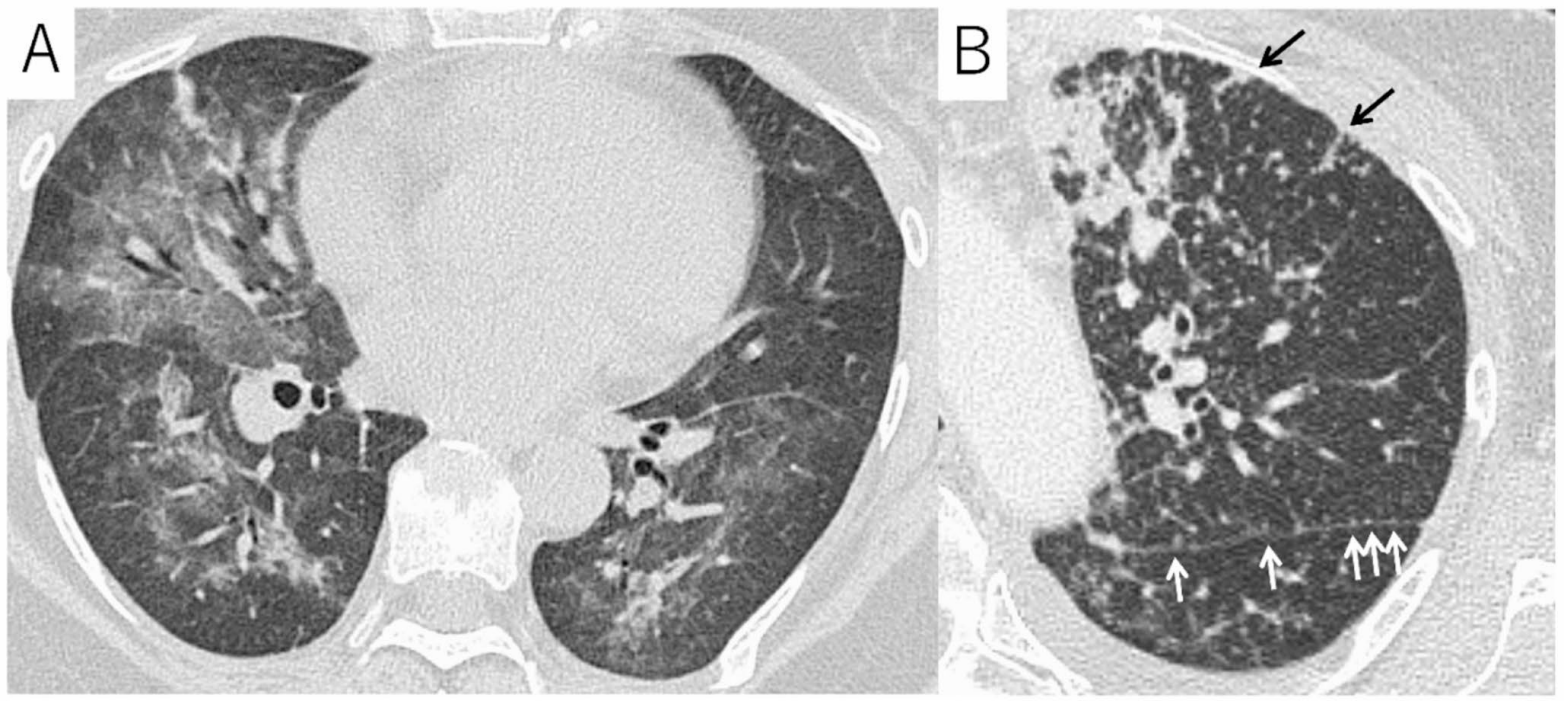
Differential diagnosis of drug-associated interstitial lung disease (DILD). **A** Pneumocystis pneumonia in a 56-year-old woman with breast cancer. A High-resolution CT (HRCT) shows bilateral extensive ground-glass opacities with peribronchovascular predominance. Although DILD was initially suspected, serum β-D glucan levels were elevated, and the patient was diagnosed with pneumocystis pneumonia. **B** Lymphangitic carcinomatosis in a 57 years-old woman with breast cancer. HRCT image shows multiple large nodules and micronodules. The micronodules extend along the interlobular septa and pleura, and beaded thickenings of the interlobular septa are also seen (arrows), suggesting lymphatic spread

The most important differential diagnosis. In immunocompromised patients, pneumonia caused by bacteria, fungi, viruses, and atypical pathogens should always be considered. When segmental/lobar consolidations are seen, bacterial pneumonia should be considered, when diffuse GGO or faint granular opacities are seen, pneumocystis pneumonia (Fig. 2A) or cytomegalovirus pneumonia should be considered as differential diagnoses. When multiple GGO or consolidations are seen, other viral pneumonia or atypical pneumonia should be considered as differential diagnoses. If cavitary nodules, and tree-in-bud (TIB) signs are seen mycobacterial and fungal pneumonia should be considered. In patients with hematological malignancy or immunodeficiency, it has been reported that TIB sign is frequently seen in infection, and thickening of interlobular septa is frequently seen in DILD on HRCT [19, 20]. However, differentiation from DILD on CT can be difficult. In that case, microbiological tests such as bronchoalveolar lavage (BAL) and sputum culture are essential, and early consultation with a respiratory specialist would be needed [7].

#### Lymphangitic carcinomatosis

A condition where cancer cells infiltrate pulmonary lymphatics, recognized as progression of the primary disease. HRCT shows nodular or beaded thickening of the interlobular septa and bronchovascular bundles (Fig. 2B). It is often accompanied by pleural effusion and mediastinal/hilar lymphadenopathy. Unlike DILD, the basic lung architecture tends to be relatively preserved [16].

#### Radiation pneumonitis/radiation recall pneumonitis

A history of chest radiation therapy is the key to differentiation. Typical radiation pneumonitis appears as well-demarcated consolidation or GGO corresponding to the radiation field. These are described in more detail in later chapters.

#### Pulmonary edema

Pulmonary edema can also be a differential diagnosis, as HRCT shows GGO and interlobular septal thickening. Drug-induced cardiotoxicity can also cause pulmonary edema.

### DILD by breast cancer therapeutic agents

The drugs used in breast cancer treatment are diverse, and each has a different incidence and characteristics of DILD.

#### Molecular targeted therapies

##### CDK4/6 inhibitors (abemaciclib, palbociclib, ribociclib)

**Fig. 3:**
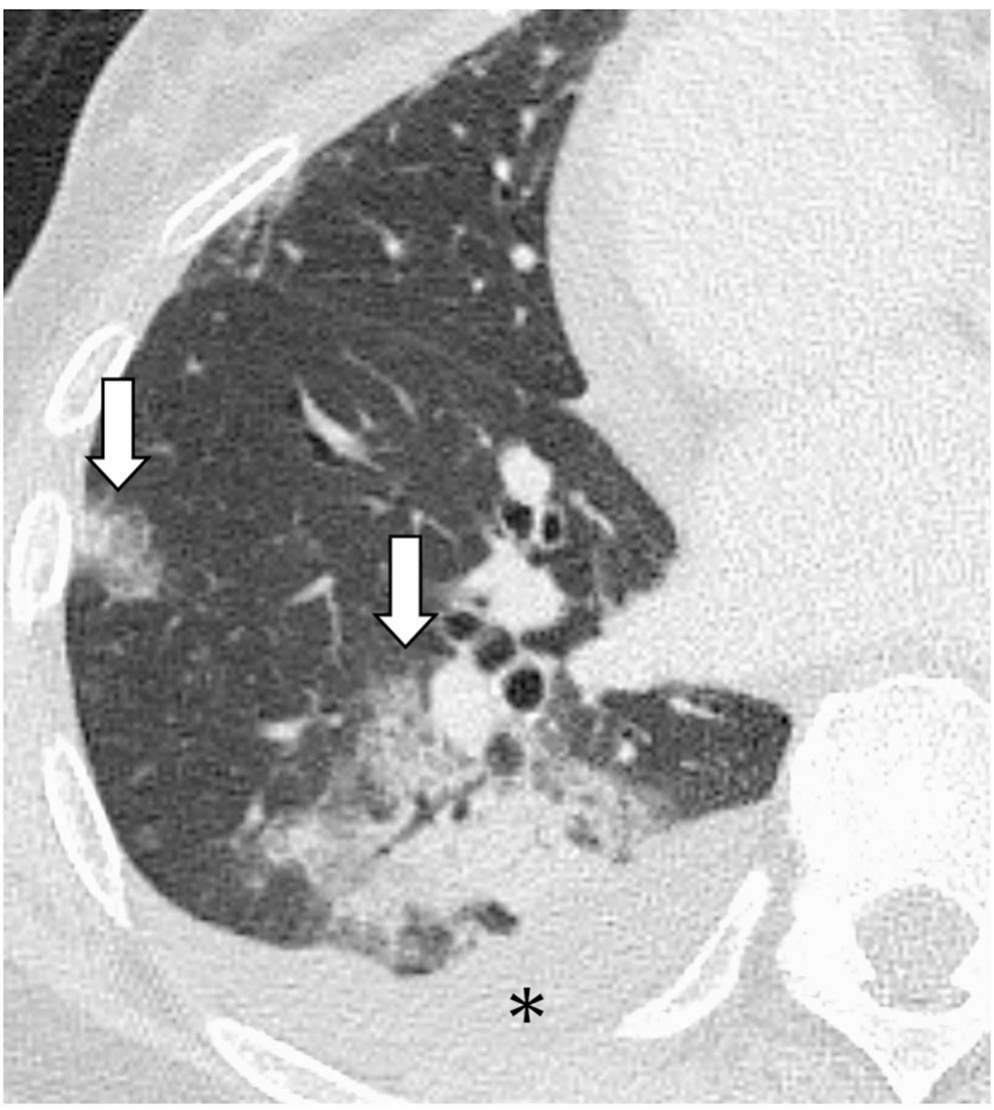
Abemaciclib-related pneumonitis in a 76-year-old woman. The patient, who had multiple pulmonary, pleural, and bone metastases from luminal type breast cancer, was receiving letrozole and abemaciclib. High-resolution CT (HRCT) image shows multiple ground-glass opacities and consolidation in the right lower lobe of the lung (arrows). Drug-associated interstitial lung disease with organizing pneumonia (OP) pattern due to abemaciclib was suspected. After discontinuation of abemaciclib, the opacities subsequently improved. Right pleural effusion (*) is also seen

Abemaciclib (Fig. 3): DILD of Grade 1–2 was reported in 2.7% of patients [5]. In post-marketing surveillance in Japan, it was reported that among an estimated 4700 users in the first year after launch, 82 cases (1.7%) of interstitial pneumonia-related events occurred, with 13 deaths, reaching a mortality rate of about 16% in DILD cases. Advanced age and a DAD pattern were identified as potential risk factors for cases with poorer outcomes [21]. In recent real-world surveillance in Japan, incidence of abemaciclib related DILD was reported in 5% (*n* = 59/1189) and mortality rate was 0.7% (*n* = 8/1189) [22].

Palbociclib: DILD was reported in 3% of patients [23].

Ribociclib: DILD was reported in 1.5% of patients [5, 23].

No specific imaging pattern has been established for each drug. DAD pattern was pointed out to be predominant in fatal cases. Other patterns such as OP, GGO-dominant, HP and NSIP patterns have also been reported [21, 23].

##### mTOR inhibitors (everolimus)

Everolimus, an mTOR (mammalian target of rapamycin) inhibitor, is one of the treatment options for HR-positive advanced/recurrent breast cancer, but it is known for its high incidence of DILD (3–33%), however its prognosis is relatively good [24–27]. The most common imaging patterns are OP, HP and NSIP patterns [25, 28].

##### Anti-HER2 agents (trastuzumab deruxtecan; T-DXd)

**Fig. 4:**
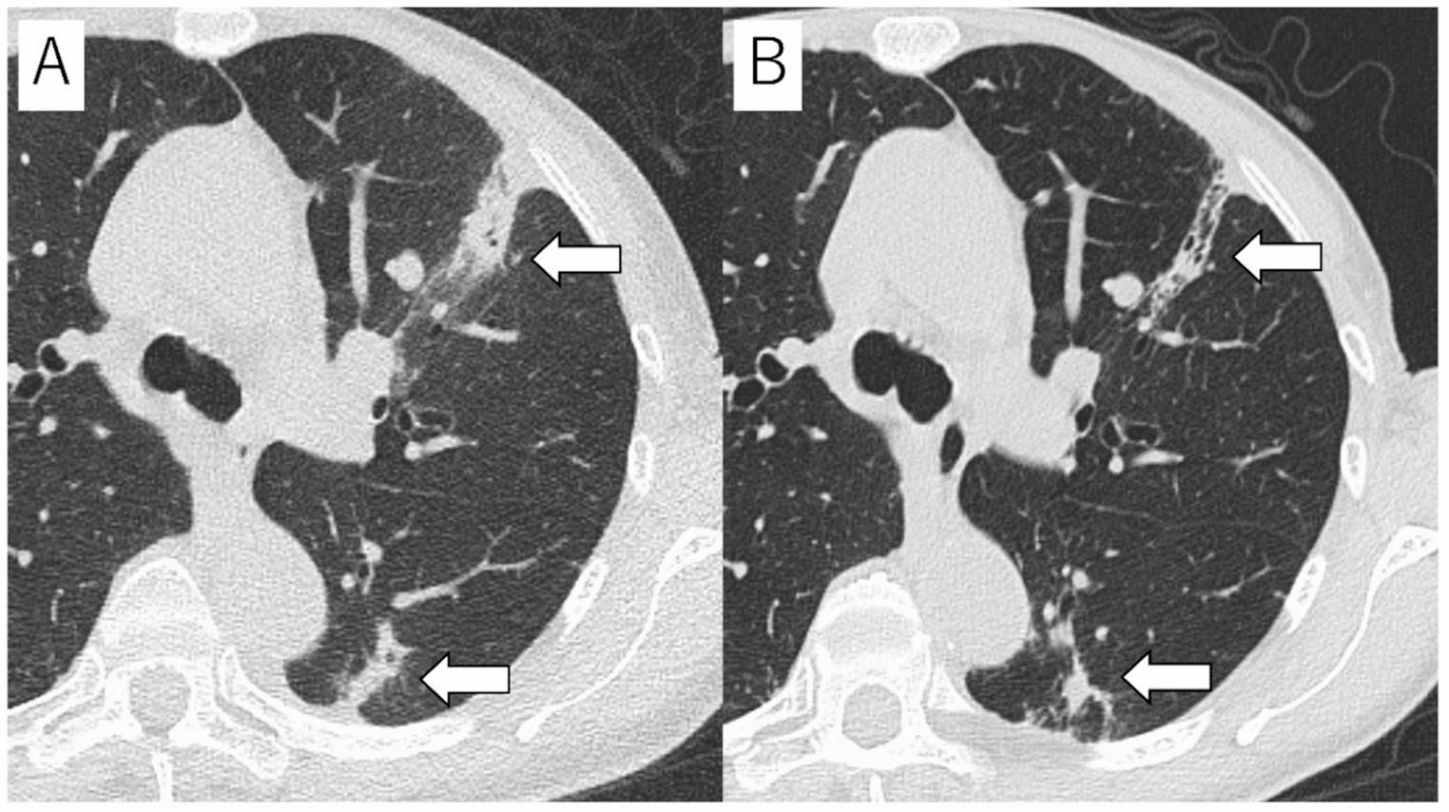
High-resolution CT (HRCT) images of Trastuzumab Deruxtecan (T-DXd)-related pneumonitis in a 76-year-old woman. The patient had multiple lung metastases from luminal HER2 type breast cancer, and was receiving T-DXd. HRCT image shows multiple consolidations in the left upper and lower lobes of the lung (arrows) (**A**). Drug-associated interstitial lung disease with an organizing pneumonia (OP) pattern due to T-DXd was suspected. Since the patient was asymptomatic, the severity is classified as grade 1 according to the Common Terminology Criteria for Adverse Events (CTCAE), and treatment was temporarily discontinued. A follow-up HRCT image obtained two months later shows decreased consolidation (arrows) (**B**), and there were no further changes, so administration was resumed at a reduced dose

Trastuzumab Deruxtecan (T-DXd) is an Antibody-drug conjugate (ADC) in which the anti-HER2 antibody trastuzumab is linked to the topoisomerase I inhibitor deruxtecan via a linker, and it shows high efficacy against HER2-positive breast cancer [29, 30]. However, DILD is the most notable serious adverse event. The incidence of all-grade ILD/pneumonitis is reported to be around 10–15% across various studies, with Grade 3 or higher events occurring in approximately 1–3% and Grade 5 (fatal) events in 0.5–1.5% [29, 30]. The exact mechanism remains unclear, but it is thought to involve a combination of factors including the direct cytotoxicity of the payload, a topoisomerase I inhibitor (deruxtecan derivative), to alveolar epithelial cells and alveolar macrophages (including bystander effects), immune-mediated responses to the drug components, and release of inflammatory cytokines [30]. While no single strong predictor has been established, potential risk factors under discussion include Japanese ethnicity, pre-existing lung disease, impaired renal function, older age, and smoking history [30]. The median time of onset of DILD is about 4 to 6 months after the start of treatment, but it can also occur earlier or later. Early symptoms are mainly cough (dry cough) and dyspnea, and may be accompanied by fever and fatigue. In some cases, the disease is asymptomatic and may be discovered only through imaging findings [29, 30]. HRCT has been reported to show the most common pattern of OP (Fig. 4), but it can also show a variety of patterns, including NSIP, HP, and DAD patterns [30, 31]. DAD pattern is seen in approximately 15% of cases of DILD [32]. If respiratory symptoms appear during T-DXd treatment or if abnormalities are found during regular imaging monitoring, ILD/pneumonia should be suspected immediately and differentiated from other diseases such as infection. A multidisciplinary team approach is critical.

##### Other anti-HER2 therapies (trastuzumab, pertuzumab, trastuzumab emtansine)

The risk of DILD with anti-HER2 therapies other than T-DXd is considered relatively low [33–37].

##### Cytotoxic agents (paclitaxel and docetaxel)

The reported incidence of DILD varies widely, from 0.7 to 12% at Paclitaxel and 7 to 26.5% at docetaxel [33]. These microtubule inhibitors are crucial in early and advanced breast cancer. Paclitaxel can cause acute hypersensitivity reactions, which may involve bronchospasm [38]. Docetaxel has reported the interstitial pneumonitis with OP pattern [39].

##### Anthracyclines (doxorubicin, epirubicin)

Widely used in breast cancer, anthracyclines are primarily known for their cardiotoxicity. While infrequent, DILD has been reported, however many are in the context of regimens combined with cyclophosphamide and 5-FU, making it difficult to identify which drug is the true cause [33, 40, 41]. The risk may be increased with concurrent thoracic radiation [38]. Rare cases presenting as HP or OP patterns have been reported [40, 41].

##### Immune checkpoint inhibitors (ICIs)

ICIs such as anti-PD-1 and anti-PD-L1 antibodies (Pembrolizmab and Atezolizmab) are used for triple-negative breast cancer [5, 42]. DILD caused by ICIs is recognized as one of the immune-related adverse events (irAEs). The incidence of all-grade pneumonitis is generally in the range of 1–6%, with severe (Grade ≥ 3) cases being less common (1%) but potentially life-threatening [5, 16, 43]. Radiologic findings are diverse. The most common pattern is the OP pattern, and NSIP, HP and DAD pattern also seen [16, 44]. Bronchiolitis and sarcoid reaction (mediastinal and hilar lymphadenopathy, pulmonary nodules with perilymphatic distribution) are also seen [44]. There is also a report that FDG-PET/CT was useful for detecting irAE in various organs [45].

##### Endocrine therapy agents

Tamoxifen and aromatase inhibitors (anastrozole, letrozole, etc.) are used for long-term standard treatment of HR-positive breast cancer. It is very rare for these drugs alone to cause DILD, with reports mainly at the case report level. Estrogen has been occasionally associated with DILD, including eosinophilic pneumonia (EP) pattern [46]. In aromatase inhibitors, DILD of OP pattern have been described [47, 48].

## Radiotherapy-associated lung injury (RLI)

Radiation therapy (RT) is established as an indispensable element in achieving local-regional control in the curative treatment of breast cancer, both after breast-conserving surgery and post-mastectomy [49–51]. In recent years, in addition to conventional irradiation methods, there has been an increasing trend in reports on hypofractionated radiotherapy, and there are also reports on intraoperative radiotherapy and carbon-ion radiotherapy [50, 52, 53]. However, among the potential adverse events associated with thoracic irradiation, radiotherapy-associated lung injury (RLI) stands as one of the most critical dose-limiting toxicities [54]. RLI rarely can significantly impair a patient’s QOL. RLI is not a single event but a dynamic process that unfolds over time, generally divided into two distinct phases: an acute phase of RLI and a subsequent chronic phase of radiation fibrosis (RF) [3, 55, 56]. RLI usually occurs within 6 months after RT (most often within 12 weeks). Radiation exposure increases capillary permeability, resulting in pulmonary edema. Damage to type I and type II pneumocytes leads to loss of surfactant and leakage of serum proteins into the alveoli. These mechanisms are thought to cause RLI [3, 55], and then leads to fibrosis (RF). RF starts to occur between 6 and 12 months after RT [55, 56]. Radiation-induced OP, which is OP secondary to RT, autoimmune mechanisms are thought to be involved in the development [57, 58]. In addition to the lungs, RT for breast cancer can also damage to the heart and skin [59, 60].

### Clinical symptoms

The clinical symptoms of RLI are non-specific and typically include a persistent dry cough, exertional dyspnea, low-grade fever, and general fatigue [55, 56]. However, it is clinically crucial to distinguish between radiological RLI which is asymptomatic and clinical RLI which is symptomatic. The severity of RLI is classified using a standardized system such as the CTCAE grade or Radiation Therapy Oncology Group (RTOG) criteria [3, 55].

### Incidence and risk factor for RLI

The reported incidence of RLI varies significantly depending on the study design and evaluation criteria, ranging from 1 to 80% [56, 61–63]. This wide discrepancy is also due to the difference in the incidence of radiological RLI (rRLI) versus clinical RLI (cRLI). The incidence of symptomatic cRLI is ranging from 0 to 64% [56] and its incidence is lower than rRLI. Risk factors for RLI include patient factors such as age, smoking history, comorbidities and the pre-existing-lung disease such as chronic obstructive lung disease (COPD) or ILD, as well as treatment-related factors such as mean lung dose (MLD) and the lung volume receiving 20 Gy (V20) [3]. Combined therapy with tamoxifen and GnRH agonists, or paclitaxel are also considered risk factors for RLI [49]. Dosimetry factors and inflammation-based indexes including neutrophil-to-lymphocyte ratio and platelet-to-lymphocyte ratio have been reported as predictive factors of RLI [64, 65].

### Radiological imaging findings

Radiological abnormalities of early RLI usually appear within 4 to 12 weeks after radiation therapy [66] on CT. CT images clearly show the characteristic imaging findings of RLI. The typical CT finding is GGO or consolidation within the irradiated lung [66]. It is characterized by the absence of specific distribution and consistency with the radiation field. In the late phase, architectural distortion and volume loss are also seen, reflecting fibrosis [3]. In breast cancer cases, especially those after conservative surgery, these findings are seen in the lung just below the anterior chest wall and in the apical lung, reflecting the irradiated field.

**Fig. 5:**
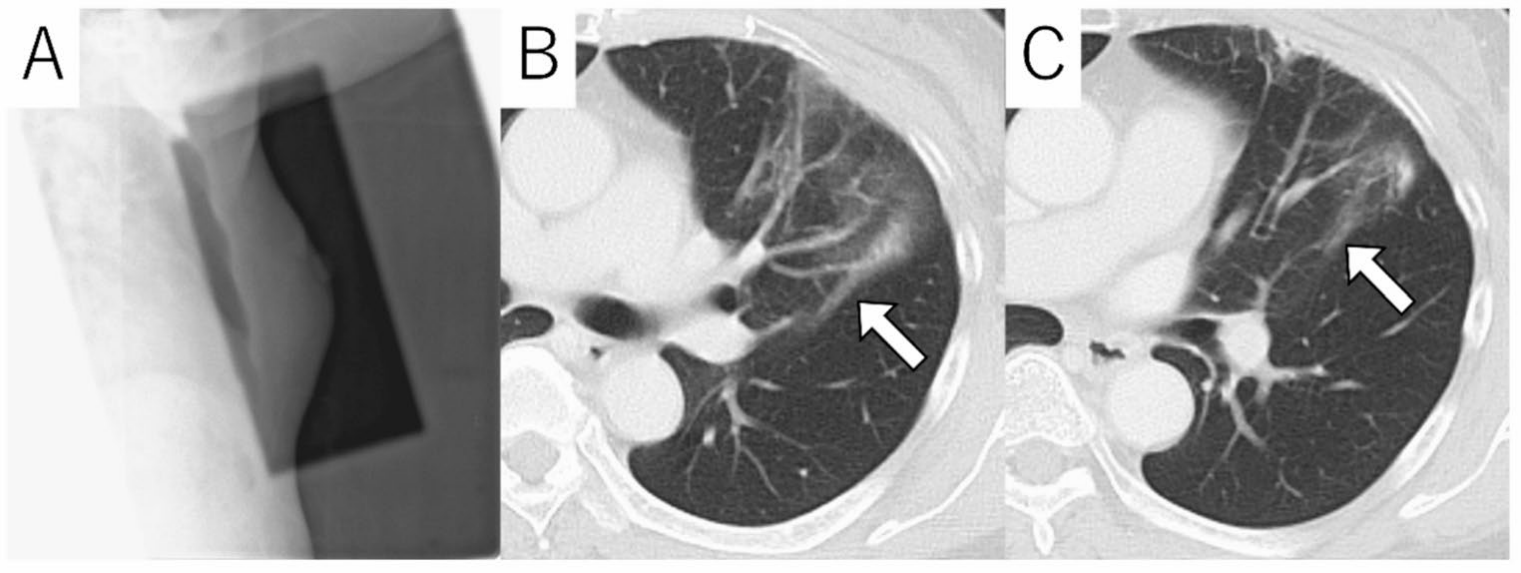
Radiation-induced organizing pneumonia (OP) in a 61-year-old with breast cancer. The patient underwent radiation therapy after breast-conserving surgery. Portal radiograph shows a tangential irradiated field (**A**). CT images show ground-glass opacities and consolidations extending beyond the irradiated field (arrows) (**B**, **C**)

Although atypical and rare, shadows may appear outside the irradiated lung field, and this is called radiation-induced OP (Fig. 5) [3, 57, 58]. The incidence is about 2%, and it occurs between 1 and 10 months after the end of radiation therapy [57, 58]. Lesions may appear unilateral or bilateral, and solitary or multiple. Imaging findings show air space consolidation with or without GGO [3, 57, 58].

### Radiation recall pneumonitis (RRP)

**Fig. 6:**
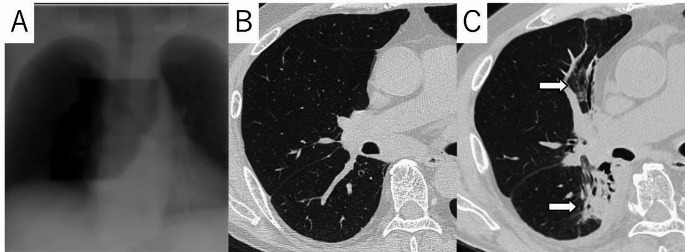
Radiation Recall Pneumonitis in a 71 years-old woman with lung cancer. Portal radiograph shows an irradiated field (**A**). High-resolution CT (HRCT) image taken one month after irradiation showed no shadows corresponding to the irradiated field. However, HRCT image taken after starting immune checkpoint inhibitor (duruvalmab) showed ground-glass opacities and consolidations (arrows) corresponding to the irradiated field. RRP was diagnosed

RRP is a unique form of RLI and is important to understand in the long-term management patient. RRP is a rare phenomenon in which an acute inflammatory response occurs in previously irradiated field of the lung, triggered by a subsequently administered drug (e.g., chemotherapy, immunotherapy) or vaccine (Fig. 6) [67–70]. Although the exact mechanism has not yet been fully elucidated, several hypotheses have been proposed, including that radiation damages lung stem cells, making them hypersensitive to subsequent drugs; that radiation increases vascular permeability, making it easier for drugs to accumulate locally; and that radiation lowers the threshold for local immune responses, causing the drugs to trigger hypersensitivity reactions [67–69]. RRP can be caused by various types of drugs, including chemotherapy drugs (taxanes, anthracyclines, gemcitabine, etc.) immune checkpoint inhibitors (nivolumab, pembrolizumab, atezolizmab, etc.), everolimus and trastuzumab, etc [67–70]. In recent years, cases of COVID-19 vaccine-related RRP have also been reported [71, 72]. The incidence of RRP varies depending on the type of triggering drug, and have reported a relatively high incidence, especially when using ICIs (7–19%) [73, 74]. Symptoms are non-specific and include cough, shortness of breath, chest tightness, chest pain, and low-grade fever [67, 69]. RRP often occurs soon after initiation of administration of a triggering drug, however may occur after several courses [69]. Imaging findings of RRP shows GGO or consolidations corresponding to the irradiated field [68, 69]. These shadows may develop or worsen months or years after radiation therapy is completed, triggered by the administration of drugs [69]. DILD and infection are considered as differential diagnoses of RRP, however the extent of the lesions in these does not match the irradiated field. Therefore, in diagnosing RRP, it is important to compare the radiation treatment plan with the portal radiograph or CT image and confirm that the shadow matches the irradiated field [69].

## Conclusion

Although advances in breast cancer treatment have significantly improved survival rates, DILD has become recognized as an important issue in clinical practice. The clinical features of DILD are extremely diverse. Various drugs can cause lung injury, but in breast cancer treatment, abemaciclib and T-DXd are important due to the high severity and frequency of DILD. Imaging findings are classified into various patterns based on HRCT findings, and the DAD pattern suggests the aggravation of DILD. Therefore, it is important to distinguish whether the image shows a DAD pattern or not.

RLI is one of the most important dose-limiting toxicities. RLI usually occurs within the radiation field, but there is also a type of lung injury called radiation-induced OP that forms shadows outside the radiation field. There is also a unique clinical condition called RRP that develops due to the use of certain drugs long after the end of radiation therapy.

Differential diagnoses for both types of lung injuries include infection and lymphangitic carcinomatosis. A comprehensive diagnosis based on clinical findings and imaging findings is required to make this differential diagnosis. Furthermore, the development of artificial intelligence or radiomics-based software may be necessary [75, 76]. In RLI, it is also important to confirm the extent of irradiation of the lungs.
